# Personalized predictive modeling of otitis media with effusion: a multivariable approach to individualized prognosis, hearing rehabilitation, and follow-up

**DOI:** 10.1007/s00405-025-10006-w

**Published:** 2026-02-16

**Authors:** Joan Lorente-Piera, Leticia Catasús, Francisco Javier Cervera-Paz

**Affiliations:** 1 Department of Otorhinolaryngology, Clínica Universidad de Navarra, Pamplona, Spain; 2 School of Medicine, Universidad de Navarra, Pamplona, Spain

**Keywords:** Otitis, Hearing loss, Personalized medicine prognostic analytics, Language development

## Abstract

**Introduction:**

Otitis media with effusion (OME) is a highly prevalent condition in pediatric patients, affecting up to 55% of children under two years of age. Several factors contribute to its development, including immature Eustachian tube function, allergic factors, and adenoidal obstruction, making treatment optimization challenging yet essential due to the potential consequences of this condition. The primary objective of this study is to analyze the influence of various etiological, clinical, and therapeutic variables on functional auditory outcomes using a binary logistic regression model. Secondarily, the study aims to identify whether these variables are associated with adverse outcomes such as the need for hearing aids or delayed language development.

**Methods:**

This is a single-center, observational, descriptive, and retrospective cohort study conducted between 2020 and 2023, with a 12-month follow-up period. Functional outcomes, risk of complications, and their timing were analyzed, among other variables.

**Results:**

A total of 95 patients were included, with a mean age of 3.82 ± 1.94 years, predominantly male (64.21%, *n* = 61) and with bilateral involvement (85.26%, *n* = 81). A mean hearing improvement of 9.07 ± 20.07 dB was observed at the end of the follow-up. Multivariable regression analysis revealed that the PTA at 6 months is a significant predictor of long-term auditory outcomes (*p* = 0.036), while adenoidectomy acts as a protective factor (*p* = 0.042). Chronic otitis media (COM), PTA at 12 months, and persistent OME were associated with a higher risk of requiring hearing aids (*p* = 0.036, 0.009, and 0.006, respectively), while the latter two were also linked to a greater risk of delayed language development (*p* = 0.030 and 0.011).

**Conclusions:**

The study identifies that PTA at 12 months, COM, and persistent OME significantly increase the risk of needing hearing aids and speech therapy. Elevated PTA at 6 months is an early predictor of poor prognosis, while adenoidectomy, despite being a protective factor for auditory outcomes, may be associated with earlier complications. Evenmore, this work illustrates how real-world clinical and audiological data can be translated into actionable personalized care pathways, supporting more precise clinical decisions and improving long-term outcomes in children with OME.

## Introduction

Otitis media with effusion (OME) is defined as the accumulation of fluid in the middle ear without the presence of clinical manifestations of an active infection [1]. This condition is highly prevalent among pediatric patients, with rates reaching approximately 55% in children under two years of age [2, 3]. Often, children in this age range may present with OME alongside recurrent episodes of acute otitis media (AOM), and it is common for seromucous material to persist in the tympanic cavity weeks after an AOM episode [4].

Historically, the etiopathogenesis of these conditions has been associated with Eustachian tube dysfunction due to immaturity, leading to poor ventilation of the middle ear and difficulty in clearing accumulated secretions [5]. Consequently, there is a natural decline in the occurrence of these diseases over time, as observed by Maw A.R. et al. in a cohort of over 200 subjects, reflecting the natural history of OME [6]. However, many other contributing factors have been described, such as a history of preceding or underlying colds, allergic history, or gastroesophageal reflux disease [7]. Furthermore, recent studies have highlighted genetic and epigenetic factors associated with susceptibility to developing these infections [8, 9].

In children, the most common symptoms of OME include hearing loss and a sensation of ear fullness. While in many cases, this condition may resolve spontaneously, the absence of obvious signs of acute inflammation allows it to go unnoticed for an extended period. If not treated appropriately, it may lead to persistent hearing loss, potentially impacting language development and academic performance [10]. Additionally, there is a risk of progression to chronic otitis media (COM) with tympanic morphological disruptions resulting from altered pressure dynamics, including tympanic retractions, keratin retraction pockets, and, in the worst cases, the development of acquired secondary cholesteatoma [11].

Given the high prevalence and potential negative impact on patient development, it is essential to optimize and initiate treatment early. However, managing OME remains a challenge as consensus guidelines on medical or surgical interventions are primarily based on indirect evidence. These recommendations, often generalized, are not always consistently applied in clinical practice. Regarding surgical intervention, the insertion of transtympanic ventilation tubes (TVTs) is considered the gold standard. It has been reported as an effective strategy to improve hearing loss and slow the progression of tympanic alterations, though it can cause persistent perforation or tympanosclerosis scars post-insertion [12].

Although significant work, such as the International Consensus on OME Management proposed by Simon et al., emphasizes the benefits and indications of tympanostomy tubes [13], the underlying hypothesis of this study is whether various factors—including the disease’s etiopathogenesis, clinical characteristics, duration, and surgical procedures—affect outcomes following TVT insertion. In recent years, personalized medicine has emerged as a paradigm shift in pediatric otolaryngology, where clinical decisions are increasingly guided by individualized risk profiles rather than population-based averages. However, in OME — one of the most prevalent conditions in childhood — there is still a lack of integrated, data-driven models to personalize prognosis and management. Our study addresses this gap by providing a multivariable model capable of predicting auditory and developmental outcomes at the individual level, offering a practical tool for precision otology. To further underline the novelty of our approach, we emphasize that the value of the present study lies not in merely re-stating well-known risk factors such as persistent effusion or elevated PTA, but in integrating longitudinal PTA data, etiological subtypes, and therapeutic interventions into a single multivariable framework. This framework aims to generate dynamic, patient-specific risk profiles that can inform early adjustments to therapy and follow-up scheduling, thereby operationalizing the principles of precision otology in routine clinical care.

Therefore, the primary objective of this study is to analyze the influence of these variables on functional auditory outcomes 12 months postoperatively. Secondarily, the study seeks to determine whether these variables are associated with adverse outcomes such as the need for hearing aids, delayed language development, or long-term complications.

## Materials and methods

### Study design

A single-center, observational, descriptive cohort study was conducted in a tertiary care hospital between 2020 and 2023. Eligible children undergoing tympanostomy-tube (TVT) insertion for OME were enrolled retrospectively at the time of surgery and subsequently followed up in the pediatric otolaryngology clinic.

#### Recruitment criteria

- Patients under 18 years diagnosed with OME, regardless of involvement of other otolaryngological structures.
- Follow-up and outcome records before and after treatment, considering each patient’s specific etiology and characteristics.
- Informed consent obtained from legal representatives in accordance with the principles of the Declaration of Helsinki (1975).
- Patients with isolated sensorineural hearing loss, genetic hearing loss and/or associated malformations were excluded.

### Diagnosis of OME

All patients underwent a physical examination, including otomicroscopic exploration. In cooperative patients, pure-tone audiometry (AC 40, Interacoustics AS, Assens, Denmark) was performed to determine air- and bone-conduction thresholds at 0.5, 1, 2, and 3 kHz averaged across both ears to obtain a single patient-level value and expressed in decibels of hearing level (dB HL). Bone-conduction thresholds were obtained with contralateral masking using narrowband noise, according to standard pediatric audiological practice, whenever age and cooperation permitted and whenever an air–bone gap or interaural difference suggested a risk of cross-hearing. Hearing loss was classified according to the Bureau International d’Audiophonologie (BIAP) criteria. Even more, tympanometric recordings were obtained (AT235, Interacoustics AS, Assens, Denmark), with a Jerger type B curve indicating effusion in these patients. In uncooperative patients, otoacoustic emission measurements were performed (ICS Chartr EP 200, Otometrics, Assens, Denmark). In cases where the need for hearing aids was suspected, a speech audiometry test was conducted using the phrase test for children proposed by Cárdenas and Marrero [14].

### Language delay

Children diagnosed with OME and suspected of language delay underwent tests to quantify their developmental level, based on the results of the Peabody Picture Vocabulary Test-III (PPVT-III). This test assesses receptive vocabulary in children, measuring their ability to understand spoken words by associating them with images [15].

Additionally, the Reynell Developmental Language Scales, a standardized test for children under 6 years of age, was used to evaluate both receptive and expressive language development. This test provides detailed information about language comprehension and production at different developmental levels [16].

In both cases, a pathological result in the questionnaires was defined as an equivalent age lower than the actual age of the subjects.

### Treatment

In patients diagnosed with OME and concomitant adenoid hypertrophy, medical treatment with montelukast and intranasal corticosteroids was administered for 12 weeks prior to considering surgical intervention [17]. An adenoid hypertrophy was considered pathological if reached grade 3 or 4 according to Parikh´s et al. endoscopic classification [18].

The insertion of transtympanic ventilation tubes (TVTs), either Donaldson or Goode type, was performed under general anesthesia via inhalation or intubation when performed simultaneously with an adenoidectomy. This treatment was chosen for patients with persistent OME despite three months of medical treatment, PTA > 30 dB, or progression of morphological changes in the tympanic membrane, as classified by Tos and Sadé [19]. Donaldson tubes were initially used in all cases, except for patients with two or more TVT insertions due to recurrence of OME, where long-term Goode TVT were employed.

Finally, hearing aids were prescribed for patients who, in the absence of active infection, had audiometric results indicating sensorineural hearing loss with an average threshold above 35 dB or a maximum speech discrimination score below 100% at 65 dB at the end of follow-up.

### Follow-up and variables

Preoperative PTA (prePTA), defined as the audiometric evaluation performed immediately before surgery and after completion of the standardized medical treatment previously indicated. Postoperative follow-up included medical visits at three months, six months, and one year after surgery, with continuous monitoring of the otological condition. Audiometric data were collected at the last evaluation before the surgical procedure and compared with results obtained one year later. Additionally, the risk of complications during the follow-up period, the time to their onset, and the need for a second TVT insertion surgery were recorded.

Demographic variables (age, sex, laterality) were also considered, as well as clinical variables such as prior surgeries, need for hearing-aid adaptation, the type of TVT used in the surgical procedure and associated etiologies as persistent OME, adenoid hypertrophy, or COM with tympanic perforation. More precisely, for the purposes of this study, COM was defined as chronic suppurative otitis media with a persistent tympanic membrane perforation and middle-ear effusion lasting more than three months, in contrast to persistent OME with an intact tympanic membrane. The study also analyzed postoperative complications, including otorrhea, early extrusion of the tympanostomy tube defined as extrusion occurring within the first 6 months after surgery, residual tympanic membrane perforation, atelectasis, at 3, 6, and 12 months and suspected labyrinthitis or inner-ear involvement, defined as a new or clearly worsened sensorineural component on pure-tone audiometry, with or without concurrent vestibular symptoms, in the absence of acute middle-ear infection. The choice of predictors was based on clinical plausibility and prior literature on OME prognosis, rather than on automated variable-selection procedures, in order to minimize overfitting in this modestly sized cohort.

### Statistical analysis

Descriptive statistical methods, including arithmetic means, standard deviations, and ranges, were used for each group before and after treatment.

To compare functional auditory outcomes at the predefined follow-up points (3, 6, and 12 months) across etiological subgroups (persistent OME, adenoid hypertrophy, or COM with perforation), we used the Kruskal–Wallis test, as the Shapiro–Wilk test indicated that several continuous variables were not normally distributed (*p* < 0.05).

A binary logistic-regression model was then developed to explore the relationship between multiple predictors and PTA at 12 months, defined as the dependent binary outcome (pathological if the mean threshold exceeded 20 dB HL, non-pathological otherwise). The 20 dB HL threshold was chosen in accordance with BIAP criteria for normal hearing, with the aim of creating a clinically meaningful binary outcome that could be readily applied for risk stratification in daily practice.

Before proceeding with the analysis, a multicollinearity test was conducted to ensure that the Variance Inflation Factor (VIF) did not compromise the model’s stability and reliability, using a VIF < 10 as the criterion. The formula for this model is as follows:

$$\begin{array}{c}In\;\left(\frac{PTA\;12M\;Pato\log ic}{1-\;PTA\;12M\;Pato\log ic}\right)=\beta0+\;\beta1+\;Age+\;\beta2\\\times\;PTAP\;R\;+\;\beta3\times\;PTA\:6\;M+\;\beta5\times\;Gender\\\beta6\times\;Type\;of\;TVT+\;\beta7\times\;COM+\;\beta8\\\times\;OME\;Persistent+\;\beta9\times\;Adenoidectomy+\;\in i\\\\\begin{array}{c}\\\\\begin{array}{c}\\\\\\\\\\\end{array}\end{array}\end{array}$$

In which:

**Dependent Variable**:

- **PTA 12 M Pathological**: If the result is > 20 dB, it is classified as pathological.
- **1-PTA 12M**: If the result is < 20 dB, it is classified as non-pathological.

**Independent Variables**:

- **β₀**: Constant or intercept term of the model.
- **β₁ - β₉**: Coefficients associated with each independent variable.
- **Age**: Patient’s age.
- **Pre-PTA**: PTA level prior to treatment (in dB).
- **6 M-PTA**: PTA level at 6 months (in dB).
- **3 M-PTA**: PTA level at 3 months (in dB).
- **Gender**: Male or female.
- **Type of Tubes**: Type of drainage tubes (Donaldson or Goode).
- **COM**: Presence/absence of COM with perforation.
- **Persistent OME**: Diagnosis of persistent serous otitis media lasting > 3 months.
- **Adenoidectomy/Adenoid Hypertrophy**: Whether the patient underwent adenoidectomy for an associated obstructive adenoid syndrome.
- **ϵi**: Residual error of the model.

To evaluate the association of these variables with secondary outcomes such as the need for hearing-aid use or the development of language delay, we applied the Mann–Whitney U test for continuous variables and the chi-square test of independence for categorical variables. Logistic regression was retained for binary endpoints because it does not require normally distributed predictors.

Finally, to analyse the time-to-complication onset across the etiological subgroups (persistent OME, adenoid hypertrophy, and COM with perforation), we used a Kaplan–Meier survival model with the Log-rank (Mantel–Cox) test to compare survival curves. The event was defined as the first post-operative complication requiring medical or surgical intervention during follow-up. Children who did not experience any of these complications by the end of the 12-month observation period were considered right-censored at their last follow-up visit (52 weeks).

The normality of data distribution was evaluated using the Shapiro-Wilk test. A p-value < 0.05 was considered indicative of statistical significance. Statistical analyses were performed using RStudio, version 4.3.3 (Boston, Massachusetts, USA).

## Results

### Population

From 2020 to 2023, 95 pediatric patients meeting the previously described inclusion criteria were treated surgically for OME in the pediatric otolaryngology area of our institution. Of the total 9,216 children evaluated during this time interval, this corresponds to 10.42% of the patients within this age range. The mean age of our sample was 45.87 ± 23.22 months, or 3.82 ± 1.94 years, with a predominance of males (64.21%, *n* = 61). Regarding the affected side, there was a clear predominance of bilateral cases in all patients. A summary of the demographic data is presented in Table 1.

**Table 1:** Summary of demographic data. TVT: transtympanic ventilation tubes

DEMOGRAPHIC DESCRIPTION		
**Age at diagnosis**	3.82 ± 1.94 years (6 months-12 years)	
**Gender**	34 (35.79%) Women	61 (64.21%) Men
**Affected ear**	95 Bilateral (85.26%)	
**Previous TVT surgery**	24 Yes (25.26%)	71 No (75.43%)

All patients included in the study were diagnosed with OME. Among them, a total of 72 patients (75.79%) met the criteria for persistent otitis media with effusion [1], 47 (49.47%) had accompanying adenoid hypertrophy, and 6 patients (6.31%) had chronic otitis media with tympanic perforation concomitant with middle ear effusion.

**Fig. 1:**
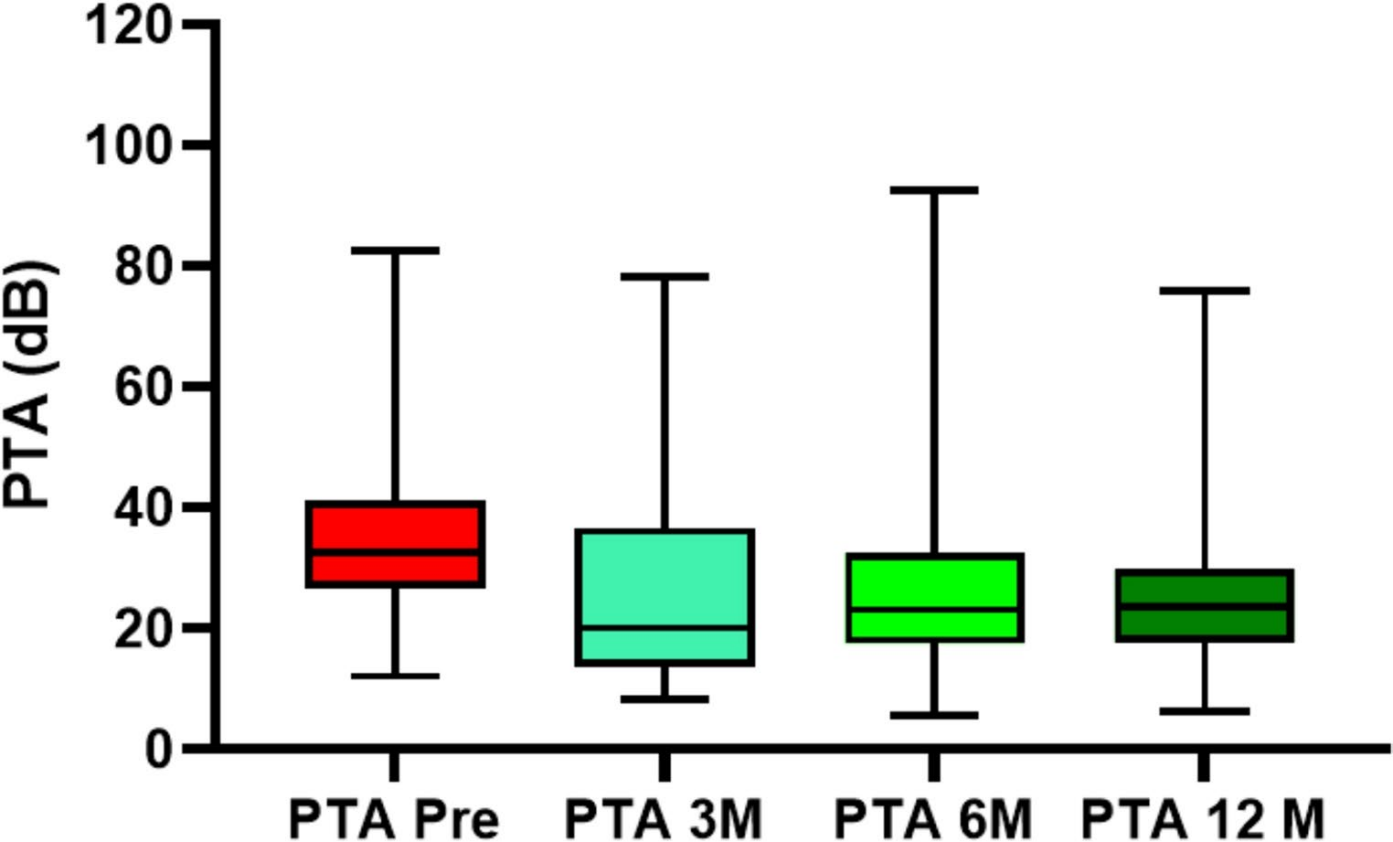
Average PTA results at each follow-up point. Pre: Pre-intervention

**Fig. 2:**
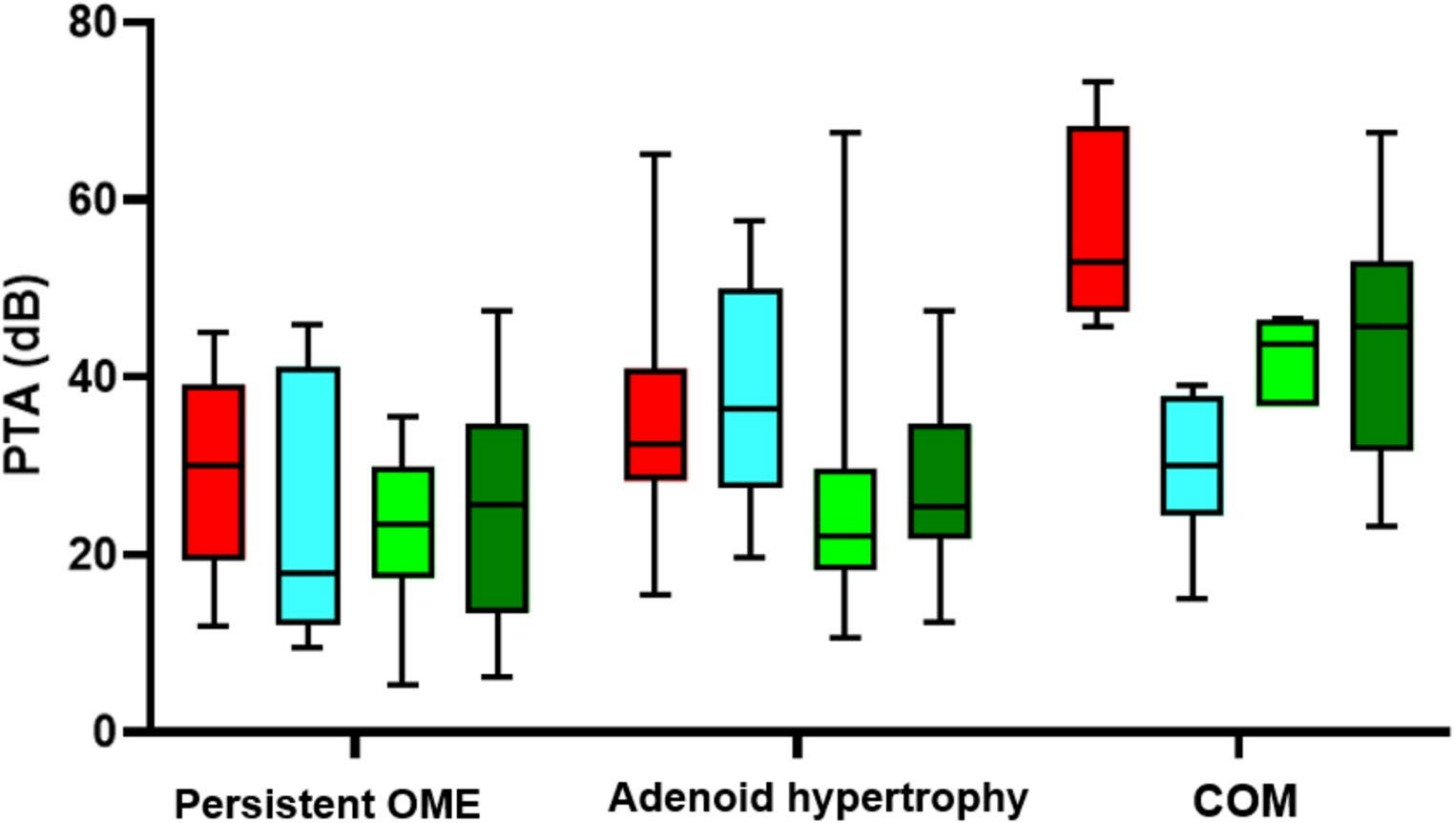
PTA evolution at different follow-up points based on the diagnosis. OME: Otitis media with effusion; COM: Chronic otitis media

When analyzing hearing loss, which was evaluated at the four predefined time points, it was found that the preoperative PTA averaged 34.95 ± 14.28 dB. Three months after surgery, it decreased to 26.11 ± 16.50 dB, remained at 26.96 ± 17.73 dB at six months, and ultimately declined to 25.88 ± 14.10 dB one year postoperatively. In other words, throughout the follow-up, there was an improvement trend of up to 9.07 ± 20.07 dB in average hearing thresholds, but the cohort still exhibited mild hearing loss. The evolution is illustrated in Figure1 and 2.

**Fig. 3:**
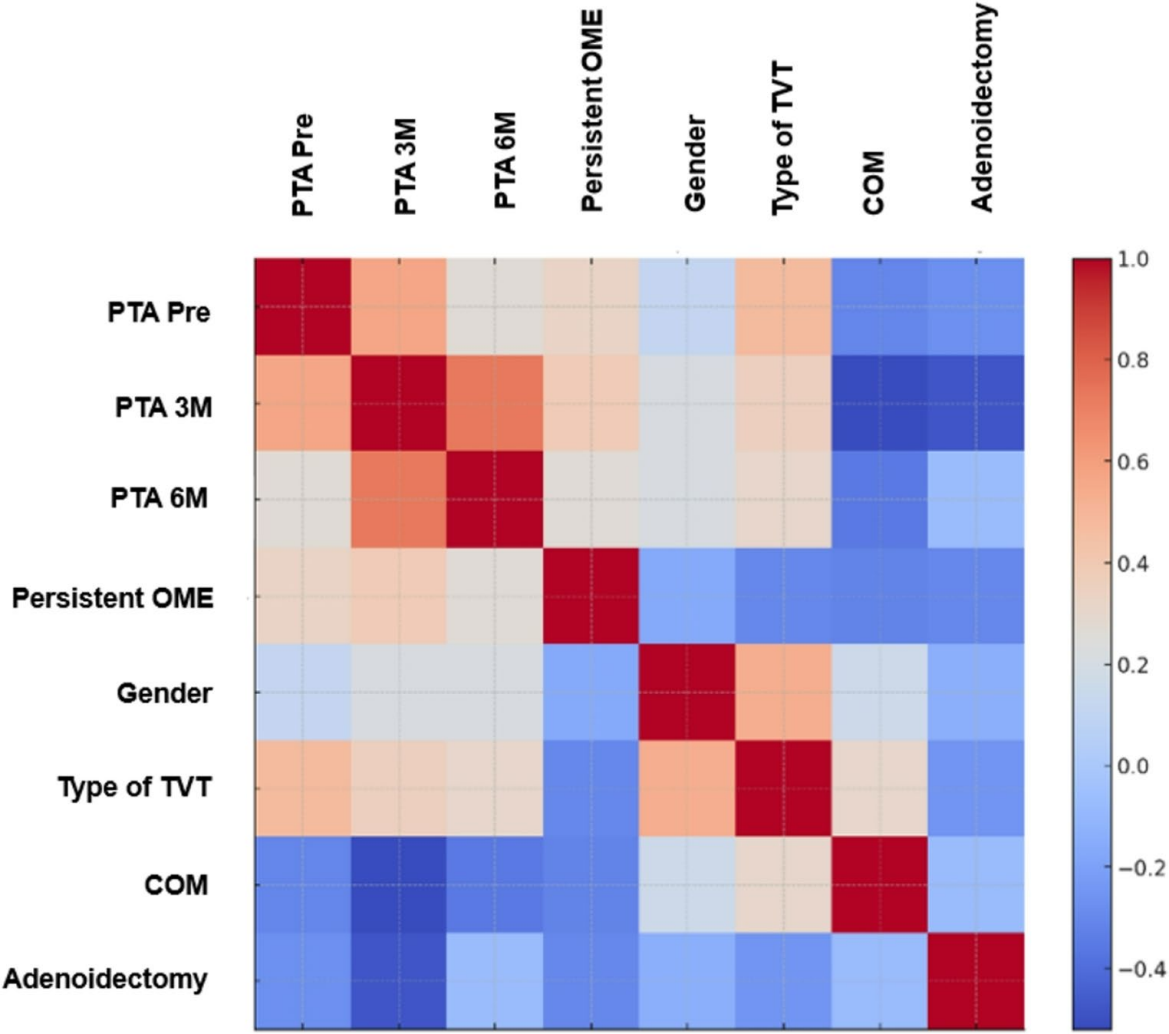
Correlation matrix among the independent variables in the applied formula. The right column shows the correlation coefficients, with 1.0 (dark red) indicating perfect positive correlation and − 1.0 (dark blue) indicating perfect negative correlation. TVT: Transtympanic ventilation tubes; OME: Otitis media with effusion; COM: Chronic otitis media

Among the treatments used, Donaldson-type transtympanic ventilation tubes (TVTs) were the most frequently employed, accounting for 91.57% of cases (*n* = 87), with the remaining 8 cases involving long-term Goode-type TVTs. Additionally, due to obstructive adenoid syndrome, adenoidectomy was performed in 47 patients (49.47%), encompassing all subjects with obstructive adenoid hypertrophy. Finally, of the 6 patients with the diagnosis of COM previously defined, only one underwent a type I tympanoplasty, as the other patients were under 8 years old and exhibited immaturity of the Eustachian tube. Now, when analyzing hearing loss based on the etiology, the results are displayed in Table 2; Figure3.

**Table 2:** PTA results at each follow-up point based on the diagnosis. *Indicates a statistically significant level, p<0.05. OME: Otitis media with effusion. COM: Chronic otitis media

**DIAGNOSIS**	**PTA Pre**	**PTA 3 Month**	**PTA 6 Month**	**PTA 12 Month**
**Persistent OME**	35.54± 35.54	19.35±16.82	26.55± 14.50	25.55± 15.66
**Adenoid hypertrophy**	29.79± 14.30	34.79± 16.53	22.78± 23.84	26.49± 11.49
**COM + Perforation**	56.20± 11.94	31.96± 5.38	42.99± 5.49	43.92± 6.69
**p value**	0.023*	0.368	0.031*	0.044*

### Functional prognosis

When analyzing the functional outcome at the end of follow-up, that is, at 12 months, it is important to assess whether PTA values at this point were within the normal range (below 20 dB) or pathological (> 20 dB). We found that 18.09% (*n* = 17) of the patients were at risk of having a pathological value at the completion of follow-up, as described in Table 3.

**Table 3:** Clinical characteristics of the groups with PTA <20 dB and >20 dB

**Independent** **variables**	**PTA <20 dB** **(n=78)**		**PTA >20 dB** **(n=17)**	
**PTA Preintervention (dB)**	28.67± 10.28		44.39± 17.90	
**PTA 3M (dB)**	24.11± 17.38		33.71± 20.74	
**PTA 6M (dB)**	22.11± 6.59		41.22± 24.33	
**Gender (%)**	35.89 Women	64.11 Men	35.29 Women	64.71 Men
**Type of TVT (%)**	94.87 Donaldson	5.13 Goode	76.47 Donaldson	23.52 Goode
**COM (%)**	0 (0%)		6 (35.29%)	
**Persistent OME (%)**	61 (78.21%)		11 (64.71%)	
**Adenoidectomy (%)**	37 (47.44%)		9 (52.94%)	
**Age (years)**	4.09± 2.06		3.79± 2.65	

To evaluate the role of the various independent variables on the functional outcome (dependent variable), we performed a binary regression model after ruling out multicollinearity (VIF < 10 for all variables). The correlation matrix is shown in Fig. 3, and the statistical significance values and coefficients are detailed in Table 4.

**Table 4:** Results of the relationship between variables and PTA at 12 months. TVT: Transtympanic ventilation tubes; OME: Otitis media with effusion; COM: Chronic otitis media. *Indicates a statistically significant level, p<0.05. Donald: Donaldson

**Independent** **variables**	**Value**		**Coefficient**	**CI (95%)**	**p value**
**PTA Preintervention**	34.95± 14.28		1.155	(-0.585-2.895)	0.387
**PTA 3M (dB)**	26.11± 16.50		1.100	(-0.440-2.641)	0.357
**PTA 6M (dB)**	26.96± 17.73		8.654	(2.103 -15.205)	0.036*
**Gender (%)**	35.79 Women	64.21 Men	2.500	(-1.640-6.644)	0.231
**Type of TVT (%)**	91.57 Donald.	8.42 Goode	1.100	(-1.101-3.306)	0.333
**COM (%)**	6 (6.31%)		16.755	(4.222-29.312)	0.242
**Persistent OME (%)**	72 (75.79%)		0.778	(-1.955-3.506)	0.899
**Adenoidectomy (%)**	47 (49.47%)		-8.965	(-14.504- -3.308)	0.040*
**Age (years)**	3.82± 1.94		0.256	(-2.804-3.316)	0.999

As we can observe, statistically significant values were only obtained for PTA at 6 months of follow-up and in cases where an adenoidectomy was performed. This suggests that the former may act as a risk factor, while the latter, with a negative coefficient, could be a protective factor.

### Risk of requiring hearing aids

Next, when analyzing the risk of these patients developing sensorineural impairment requiring hearing aids, the same independent variables previously applied to the model were reanalyzed, adding PTA at 12 months as an additional variable.

In this context, it is worth noting that 6.31% of the cohort, or 6 children, eventually required hearing aids. All were diagnosed with persistent OME, and two of them had undergone adenoidectomy for adenoidal obstruction. Furthermore, four of the six patients required multiple surgical procedures. Initially, the preoperative PTA for these subjects was 63.71 ± 17.01 dB, which decreased to 55.00 ± 18.27 dB after one year of follow-up. This represents an improvement of 8.71 ± 17.65 dB, although their hearing remained within the range of moderate hearing loss.

The results for the different variables in this analysis are presented in Table 5.

**Table 5:** Results for the risk of requiring hearing aids. TVT: Transtympanic ventilation tubes; OME: Otitis media with effusion; COM: Chronic otitis media. *Indicates statistically significant results (p<0.05)

**Variables**	**Value**		**p value**	**CI (95%)**	**Odds Ratio**
**PTA Preintervention (dB)**	63.71± 17.01		0.288	(0.928- 1.315)	1.104
**PTA 3M (dB)**	47.54± 16.13		0.387	(0.933 - 1.210)	1.063
**PTA 6M (dB)**	53.35± 18.66		0.476	(0.911- 1.156)	1.028
**PTA 12M (dB)**	55± 18.27		0.036*	(1.847 – 12.396)	4.771
**Gender (%)**	33.33 Women	66.66% Men	0.889	(0.307 - 2.899)	1.605
**Type of TVT (%)**	66.66 Donald.	33.33 Goode	0.999	(0.173- 9.74)	4.022
**COM (%)**	3 (50.00%)		0.009*	(2.432 – 9.875)	5.732
**Persistent OME (%)**	6 (100.00%)		0.006*	(2.012-8.126)	4.998
**Adenoidectomy (%)**	2 (33.33%)		0.999	(0.622 – 8.33)	4.545
**Age (years)**	5.46± 1.01		0.476	(0.972- 1.322)	1.141

In this case, it can be deduced that PTA at 12 months, COM, and persistent OME are statistically significant factors associated with a higher risk of hearing impairment. The other variables did not show a statistically significant impact.

### Risk of developing Language delay

Thirdly, we reanalyzed the variables from the previous model to study which factors have the greatest risk or impact on causing language delay. We found that 14 patients (14.74%) developed these sequelae secondary to middle ear effusion.

All these patients were diagnosed with persistent OME, and in 6 cases (42.86%), concomitant adenoid hypertrophy was present, for which adenoidectomy was performed. The PTA at the beginning of follow-up was 52.46 ± 20.71 dB, which decreased to 37.85 ± 20.61 dB by the end of the 12-month follow-up. This represents an improvement from moderate to mild hearing loss, with a gain of 14.61 ± 20.66 dB.

The results for this analysis are shown in Table 6.

**Table 6:** Logistic regression results for the risk of requiring hearing aids. TVT: Transtympanic ventilation tubes; OME: Otitis media with effusion; COM: Chronic otitis media. *Indicates statistically significant results (p<0.05)

**Variables**	**Value**		*p value*	**CI (95%)**	**Odds Ratio**
**PTA Preintervention (dB)**	52.46± 20.71		0.410	(0.920 - 1.285)	1.054
**PTA 3M (dB)**	42.26 ± 18.55		0.346	(0.911 - 1.190)	1.041
**PTA 6M (dB)**	40.11 ± 17.87		0.231	(0.780 - 1.054)	0.906
**PTA 12M (dB)**	37.85± 20.61		0.030*	(2.231 – 10.801)	6.122
**Gender (%)**	35.71 Women	64.29 Men	0.771	(0.812 - 2.987)	1.499
**Type of TVT (%)**	78.57 Donald.	21.42 Goode	0.999	(0.312 - 7.833)	4.100
**COM (%)**	4 (28.57%)		0.699	(0.121- 1.10)	0.566
**Persistent OME (%)**	14 (100%)		0.011*	(1.622- 4.897)	3.111
**Adenoidectomy (%)**	6 (42.86%)		0.999	(0.127 - 6.188)	3.212
**Age (years)**	4.78 ± 1.64		0.584	(0.972 - 1.457)	1.266

For this variable, it can be concluded that only PTA at 12 months and persistent OME contribute statistically significantly to the model, with adequate confidence intervals.

### Relationship between dependent variables

Subsequently, it was considered of particular interest to directly relate the three fundamental dependent variables analyzed so far: PTA at the end of follow-up, the risk of requiring hearing aids, and the risk of language delay.

The relationship between PTA at 12 months and the risk of requiring hearing aids was statistically significant, with a p-value of 0.036 in the Mann-Whitney U test. Even more, the relationship between PTA at 12 months and the risk of developing a language delay was also statistically significant, with a p-value of 0.030. Finally, the relationship between the risk of requiring hearing aids and developing language delay was also statistically significant, with a p-value of 0.024 in the chi-square test.

### Risk of complications and time to onset

**Fig. 4:**
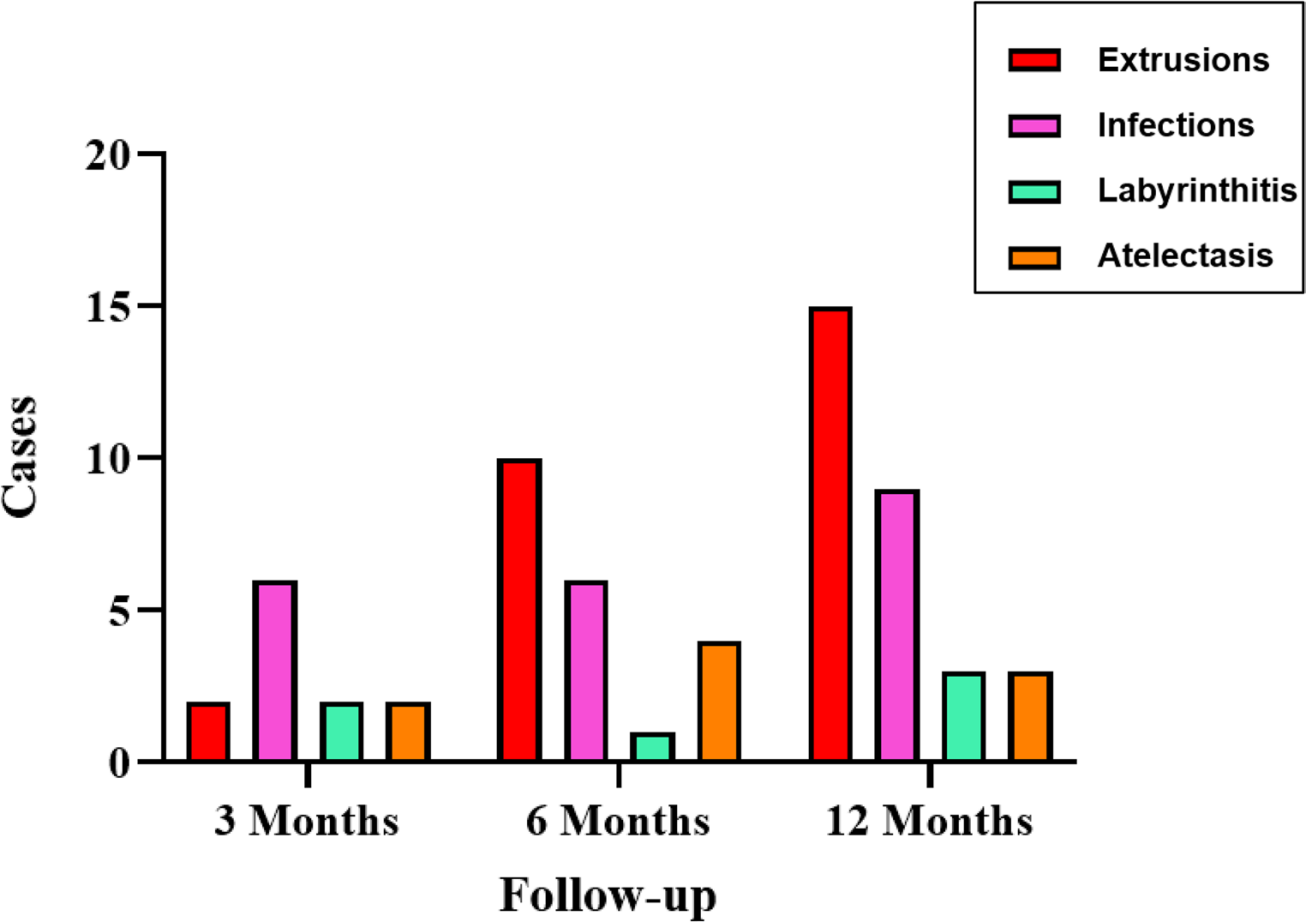
Complications observed following TVT placement at different follow-up points

When analyzing the risk of complications at different follow-up periods, we found that at three months, 12 patients (12.63%) experienced complications following TVT surgery, with acute otitis media infections being the most common. At six months, 21 patients (22.11%) experienced complications, primarily TVT extrusions. At one year of follow-up, the highest complication rate was observed, with 31.58% (*n* = 30) experiencing issues, again with tube extrusions being the most frequent complication. Only 5.26% (*n* = 5) required reintervention with the placement of new TVTs. A summary of complications by follow-up period is presented in Figure4.

**Fig. 5:**
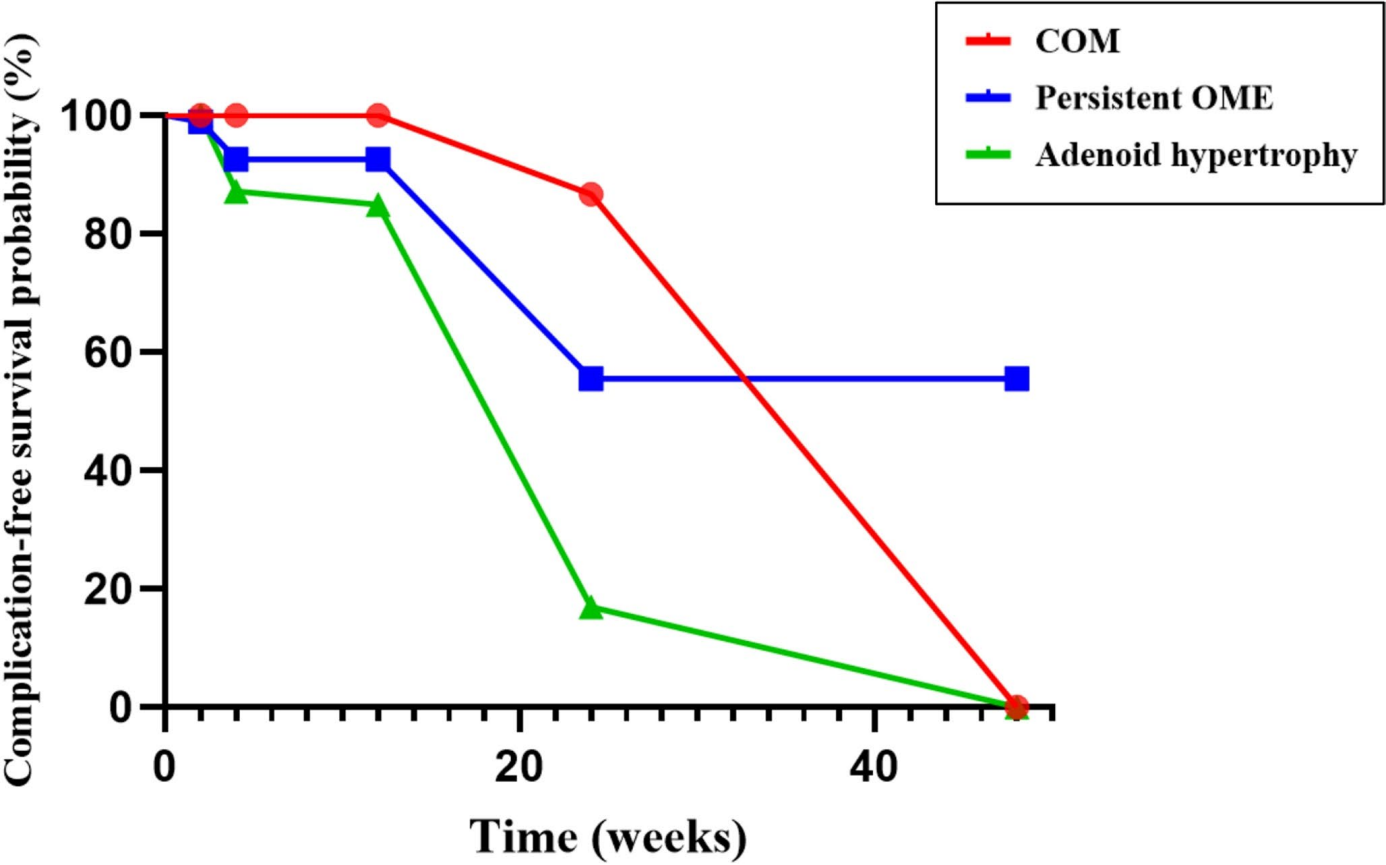
Kaplan–Meier curves showing the probability of complication-free survival over time, expressed in weeks, based on each etiological subgroup. OME: Otitis media with effusion; COM: Chronic otitis media

Finally, when analysing the time to the occurrence of clinically relevant postoperative complications according to each etiological subgroup (persistent OME, adenoid hypertrophy, and COM with tympanic perforation), the complication-free survival analysis revealed significant differences among the three groups, as shown in Figure5.

The COM group (red) showed a high initial complication-free survival, followed by a marked decline after approximately 20 weeks, reaching 0% around week 45. In contrast, the persistent OME group (blue) exhibited an early reduction in complication-free survival within the first 20 weeks, followed by a plateau without further relevant events until week 48. The adenoid hypertrophy group (green) demonstrated the steepest early decline in complication-free survival, reaching low levels by approximately week 20 and 0% near week 40. The Log-rank (Mantel–Cox) test confirmed statistically significant differences between the complication-free survival curves (χ² = 40.16, *p* < 0.0001).

## Discussion

Numerous studies in the current literature have separately examined individual factors potentially influencing functional outcomes in pediatric patients with OME. However, the present study adopts a multivariable approach to integrate these factors and explore their combined contribution to prognosis, not only from an auditory perspective but also in relation to complication risk. Rather than reiterating isolated risk factors, our aim was to assess how their weighted interaction—quantified through logistic regression—may contribute to individualized risk stratification in an exploratory framework.

One of the most clinically relevant findings is the identification of PTA at 6 months as an early dynamic marker of long-term auditory outcome. This observation supports the concept that the six-month audiometric evaluation represents a critical checkpoint in postoperative surveillance. Patients who fail to demonstrate adequate improvement at this stage are at increased risk of maintaining a pathological PTA at one year, reinforcing the need for intensified follow-up and earlier referral for speech-language intervention. This shifts the role of intermediate PTA from a merely descriptive parameter to a prognostic signal capable of guiding individualized management before long-term functional deficits consolidate.

The overall audiometric behavior of our cohort aligns with prior publications. Rosenfeld et al. [20] and the Simon et al. consensus [13] reported that most children with OME present with mild hearing loss (20–35 dB), while thresholds above 50 dB are uncommon. Our findings are consistent with these observations and further demonstrate that severe hearing loss predominantly occurs in association with chronic inflammatory pathology. In our cohort, most patients with PTA values exceeding 50 dB presented with COM, frequently associated with tympanic membrane perforation. This supports the concept that chronic middle-ear inflammation plays a major role in sustained auditory dysfunction [21–23].

The magnitude of hearing improvement observed after TVT placement in our series is also consistent with previous evidence. Systematic reviews by Browning et al. [24] and Hellström et al. [25] reported average auditory gains of approximately 10–12 dB, similar to the 9–10 dB improvement observed in our cohort over one year. This reinforces the established effectiveness of ventilation tubes in improving conductive hearing loss caused by middle-ear effusion.

Adenoidectomy emerged as a protective factor for auditory outcome at one year. This finding is in line with existing evidence supporting its role in reducing persistence and recurrence of OME [4, 26, 27], especially when combined with TVT placement [28]. The association observed in our model should not be interpreted as strictly causal, as it may reflect both mechanical relief of nasopharyngeal obstruction and a reduction in infectious burden. Interestingly, adenoidectomy was also associated with a higher rate of early postoperative complications, likely reflecting transient perioperative vulnerability rather than a detrimental long-term effect of the intervention itself.

With respect to the need for hearing aids, chronicity-related variables—persistent OME and COM with perforation—were identified as significant risk factors alongside PTA at 12 months. These conditions primarily generate conductive hearing loss; however, prolonged inflammation may promote inner-ear involvement through toxic inflammatory mediators or secondary labyrinthine irritation [29]. This mechanism may explain the transition toward mixed or sensorineural components observed in a subset of patients with chronic middle-ear disease.

Patients with COM and persistent OME exhibited the poorest long-term auditory profiles, in contrast to the relative stability observed in patients with isolated adenoid hypertrophy. This pattern may be explained by sustained inflammatory activity, persistent Eustachian tube dysfunction, and irreversible mucosal or ossicular damage, which may limit auditory recovery even after adequate middle-ear ventilation [30, 31]. It should be noted that relevant potential confounders such as socioeconomic status, environmental exposure, or parental education were not systematically recorded and may have contributed to residual confounding.

Language development was significantly associated with PTA at 12 months and persistent OME. These findings are physiologically plausible, as early language acquisition is critically dependent on stable auditory input. Even intermittent auditory deprivation, as occurs in persistent OME, may interfere with phoneme discrimination, word formation, and speech perception [32–34]. However, because baseline standardized language testing was not systematically performed in all patients, these associations should be interpreted as correlational rather than strictly causal.

Finally, the temporal distribution of clinically relevant postoperative complications differed substantially across etiological subgroups. Patients with COM with perforation exhibited a prolonged vulnerability to late complications, whereas those with persistent OME were predominantly affected in the early postoperative period. In contrast, patients with adenoid hypertrophy showed an early clustering of complications despite simultaneous adenoidectomy, likely reflecting persistent anatomical and immunological vulnerability of the Eustachian tube. These findings support the need for etiologically tailored follow-up strategies.

### Limitations

This study has certain limitations that must be considered when interpreting the results obtained. Firstly, its retrospective observational design inherently limits causal inference and prevents full control over data acquisition and variable standardization. A prospective study design would allow systematic collection of additional diagnostic variables which could further refine prognostic modeling and reduce residual confounding.

Secondly, although the overall sample size was adequate for the primary analyses, some specific subgroups—such as patients with tympanic perforation or those who developed early complications—had small sample sizes, which may have reduced the statistical power to detect significant associations in these categories. Even though a multicollinearity assessment was performed, we recognize that the inclusion of several longitudinal PTA measurements may still introduce redundancy and potentially affect the stability of coefficient estimates. Moreover, PTA at 12 months was dichotomised using a 20 dB HL cut-off. While this approach facilitated clinical interpretability of the logistic-regression model, it may have reduced statistical power and masked subtler variations in hearing outcomes.

Furthermore, for secondary endpoints such as the need for hearing aids and language delay, the events-per-variable ratio in the logistic-regression models was clearly suboptimal. These analyses should therefore be interpreted as hypothesis-generating, and the estimated odds ratios may be unstable. We did not model interaction terms because the available sample did not allow reliable estimation; such effects should be explored in larger prospective cohorts. Finally, early PTA measurements and PTA at 12 months partly capture the same underlying construct, so some degree of endogeneity and residual collinearity cannot be ruled out.

Finally, the isolated effect of preoperative medical treatment with intranasal corticosteroids and montelukast on hearing thresholds was not specifically analysed, as the study was not designed to evaluate the independent audiological impact of pharmacological therapy. All patients followed the same standardized medical protocol prior to surgery, and the preoperative PTA reflects the hearing status immediately before ventilation tube placement. Therefore, any potential contribution of medical treatment to hearing improvement could not be disentangled from the surgical effect.

## Conclusions

This study identifies PTA at 12 months, COM, and persistent OME as factors significantly associated with the risk of requiring hearing aids and the development of long-term auditory complications. Additionally, an elevated PTA at six months emerges as an early marker of poor auditory prognosis, allowing for the prediction of cases with worse outcomes. On the other hand, although adenoidectomy showed a trend toward a possible protective effect, it was also associated with the earlier appearance of complications compared to other groups. The analysis also reveals that the risk of language delay is closely related to persistent hearing deficits, reinforcing the importance of early detection and intervention.

Finally, these findings underline the need for continuous audiological follow-up and a multidisciplinary approach to reduce both the occurrence of complications and the impact on language development, thereby optimizing the functional auditory prognosis in patients with OME. By combining advanced statistical modeling with readily available clinical data, this study exemplifies the translation of personalized medicine principles into pediatric otology. Risk stratification tools like ours enable clinicians to anticipate complications, optimize hearing rehabilitation, and allocate resources efficiently, providing tailored, patient-specific care pathways for children with OME.

## Data Availability

Data pertaining to this study can be shared upon request to the corresponding author.
